# Role of Thromboelastography as an Early Predictor of Disseminated Intravascular Coagulation in Patients with Septic Shock

**DOI:** 10.3390/jcm9123883

**Published:** 2020-11-29

**Authors:** Sang Min Kim, Sang-Il Kim, Gina Yu, June-Sung Kim, Seok In Hong, Bora Chae, Yo Sep Shin, Youn Jung Kim, Seongsoo Jang, Won Young Kim

**Affiliations:** 1 Department of Emergency Medicine, University of Ulsan College of Medicine, Asan Medical Center, Seoul 05505, Korea; swdarkhorse@gmail.com (S.M.K.); jsmeet09@gmail.com (J.-S.K.); finefigs@gmail.com (S.I.H.); brchae@gmail.com (B.C.); irrusters@gmail.com (Y.S.S.); yjkim.em@gmail.com (Y.J.K.); 2 Department of Emergency Medicine, Soonchunhyang University Seoul Hospital, Seoul 04401, Korea; doctorsi0519@gmail.com; 3 Department of Emergency Medicine, University of Yonsei College of Medicine, Seoul 06273, Korea; florigen@gmail.com; 4 Department of Laboratory Medicine, University of Ulsan College of Medicine, Asan Medical Center, Seoul 05505, Korea; ssjang@amc.seoul.kr

**Keywords:** septic shock, disseminated intravascular coagulation, thromboelastography, predictor

## Abstract

(1) Background: The currently proposed criteria for diagnosing overt disseminated intravascular coagulation (DIC) are not suitable for early detection of DIC. Thromboelastography (TEG) rapidly provides a comprehensive assessment of the entire coagulation process and is helpful as a guide for correcting consumptive coagulopathy in sepsis-induced DIC. This study aimed to investigate the role of TEG in the prediction of DIC in patients with septic shock. (2) Methods: TEG was conducted prospectively in 1294 patients with septic shock at the emergency department (ED) between January 2016 and December 2019. After exclusion of 405 patients with “do not attempt resuscitation” orders, those refusing enrollment, and those developing septic shock after ED presentation, 889 patients were included. DIC was defined as an International Society on Thrombosis and Hemostasis score ≥ 5 points within 24 h. (3) Results: Of the 889 patients with septic shock (mean age 65.6 ± 12.7 years, 58.6% male), 158 (17.8%) developed DIC. TEG values, except lysis after 30 min, were significantly different between the DIC and non-DIC groups. Among the TEG values, the maximal amplitude (MA) had the highest discriminating power for DIC, with an area under the curve of 0.814. An MA < 60 indicated DIC with 79% sensitivity, 73% specificity, and 94% negative predictive value. Based on multivariable analysis, MA < 60 was an independent predictor of DIC (odds ratio 5.616 (95% confidence interval: 3.213–9.818)). (4) Conclusions: In patients with septic shock, the MA value in TEG could be a valuable tool for early prediction of DIC.

## 1. Introduction

Coagulation abnormality is an important and common complication in patients with sepsis. This dysregulation of the hemostatic system may lead to disseminated intravascular coagulation (DIC) and result in microvascular thrombosis, hypoperfusion, major organ dysfunction, and ultimately death [1,2]. Sepsis treatment currently includes early recognition of organ dysfunction and initiation of individually tailored therapy. Therefore, rapid diagnosis of DIC is essential since it facilitates sepsis management and is associated with improved outcomes [3,4]. Although the International Society on Thrombosis and Hemostasis (ISTH) has proposed criteria for diagnosing overt DIC [5], they are not suitable for early detection of DIC, including delays in the appearance of fibrin-related markers, which require tests that are typically not performed initially [6].

Thromboelastography (TEG) is a point-of-care test that quickly measures the rate (reaction time (R), clot formation speed (K), and alpha angle), strength (maximum amplitude (MA)), and stability (lysis after 30 min (LY30)) of clot formation [7]. TEG correlates with bleeding and thrombotic events and is useful for guiding hemostatic therapy in trauma [8], liver transplantation [9], and other surgeries [10]. Furthermore, it was recently reported that TEG might be valuable as an early predictor of hemorrhagic events in acute stroke [11] and favorable neurological outcomes in post-cardiac arrest care [12]. Previous studies investigating the role of TEG in diagnosing sepsis have reported that TEG is useful for identifying coagulation abnormalities and that TEG findings may be associated with clinical prognosis [13,14]. However, there is a paucity of data on the use of TEG for predicting sepsis-induced DIC in patients with septic shock [15]. The management of sepsis-induced DIC includes both treating the underlying infection and correcting the coagulopathy, with most therapeutic approaches focusing on anticoagulant therapy. We hypothesized that TEG is useful for quickly identifying patients with an increased risk of DIC at ED admission and also for guiding targeted therapy for coagulopathy. In this study, we evaluated the role of TEG as an early predictor of DIC in patients with septic shock.

## 2. Material and Methods

### 2.1. Study Design and Patients

This retrospective analysis of a prospective data registry was performed at an urban academic adult emergency department in a tertiary referral center with an annual census of more than 110,000 patients in Seoul, South Korea. Beginning in January 2010, all consecutive adult patients (aged ≥18 years) with septic shock that were diagnosed in the emergency department and treated with protocol-driven resuscitation bundle therapy were enrolled with their data prospectively collected in our institution’s Septic Shock Registry [16]. It was created for the implementation of the resuscitation bundle therapy and the monitoring tools for quality assessment of septic shock management. Patients who underwent TEG at ED admission from January 2016 to December 2019 were enrolled. The study was approved by our hospital’s institutional review board (Study No. 2016-0548), which waived the requirement for informed consent because the study involved the analysis of case registry records.

Septic shock was defined as refractory hypotension (mean arterial pressure ≤ 65 mmHg) requiring vasopressors despite adequate fluid therapy or a blood lactate concentration of at least 4 mmol/L with suspected or confirmed infection, according to previous definition [17]. The septic shock registry did not include patients with a “do not attempt resuscitation” status, those who refused to enroll in the study, or those who developed septic shock after 6 h of ED presentation. DIC was defined in accordance with the criteria outlined by the ISTH (Supplementary Table S1), i.e., an ISTH DIC score ≥ 5 [18].

### 2.2. Data Collection

Demographic and clinical characteristics, including age, sex, past medical history, source of infection, and clinical outcome (28- and 90-day mortality), were retrieved from electronic medical records of registry-enrolled patients. Blood samples were obtained within 15 min of ED presentation. Data on white blood cell counts, hemoglobin levels, blood urea nitrogen levels, creatinine levels, albumin levels, and initial lactate levels were collected. The Sequential Organ Failure Assessment (SOFA) score was calculated in the ED on initial recognition of septic shock [19]. The Acute Physiology and Chronic Health Evaluation II (APACHE II) score was calculated by using the worst parameters within 24 h of ED admission [20].

### 2.3. Thromboelastography

TEG was performed at the time of septic shock recognition. Whole blood (approximately 4 mL) was collected in vials containing citrate, by an assigned nurse. TEG was initiated with a computerized coagulation analyzer (Model 5000; Haemonetics Corporation, Braintree, MA, USA), which measured the physical properties of the clot and recorded kinetic changes. The recorded variables included R (minutes; reflects the rate of initial fibrin formation), K (minutes; reflects the clot growth kinetics), alpha angle (α; reflects the clot growth kinetics), MA (mm; reflects the clot strength), and LY30 (%; proportional reduction in amplitude after MA reflecting fibrinolysis). Subsequently, the coagulation index (CI) was calculated with the following formula: (CI = 0.1227 (R) + 0.0092 (K) + 0.1655 (MA) − 0.0241 (α) − 5.0220) [21]. The normal CI value range for humans is −3.0 to 3.0. Values > 3.0 are considered hypercoagulable, and values < −3.0 are hypocoagulable [22]. TEG software analysis (TEG Analytical Software 4.2.3; Haemonetics Corporation, Braintree, MA, USA) and all assays were performed in accordance with the manufacturer’s instructions.

### 2.4. Statistical Analysis

Continuous variables with normal distributions are represented as the mean ± standard deviation, and those with skewed distribution are represented as the median and interquartile range. Categorical variables were presented as numbers and percentages. Variables were compared to identify differences between the DIC group and non-DIC group, using Student’s *t*-test or the Wilcoxon rank-sum test for continuous variables and the chi-square test or the Fisher’s exact test for categorical variables. TEG analysis involved calculating the area under the receiver operating characteristic (ROC) curve (AUC). We determined the cutoffs for TEG values which represent an AUC more than 0.750, including K, MA, and CI, using ROC-based methods (the Youden index). To identify risk factors for the DIC, we included variables with an entry-level significance of *p* < 0.05 in the univariate analysis. After we confirmed multicollinearity with linear regression, multivariate analysis was performed by using the process of backward stepwise regression. The results of multivariate logistic regression analysis are reported as odds ratios (OR) and 95% confidence intervals (CI). In addition, standard statistical methods were used to calculate sensitivity, specificity, positive predictive value (PPV), negative predictive value (NPV), and positive and negative likelihood ratios (PLR and NLR, respectively). All statistics were performed by using SPSS software (IBM SPSS Statistics for Windows, Version 21.0, Armonk, NY, USA). A *p*-value of < 0.05 was considered statistically significant.

## 3. Results

### 3.1. Baseline Characteristics

**Figure 1:**
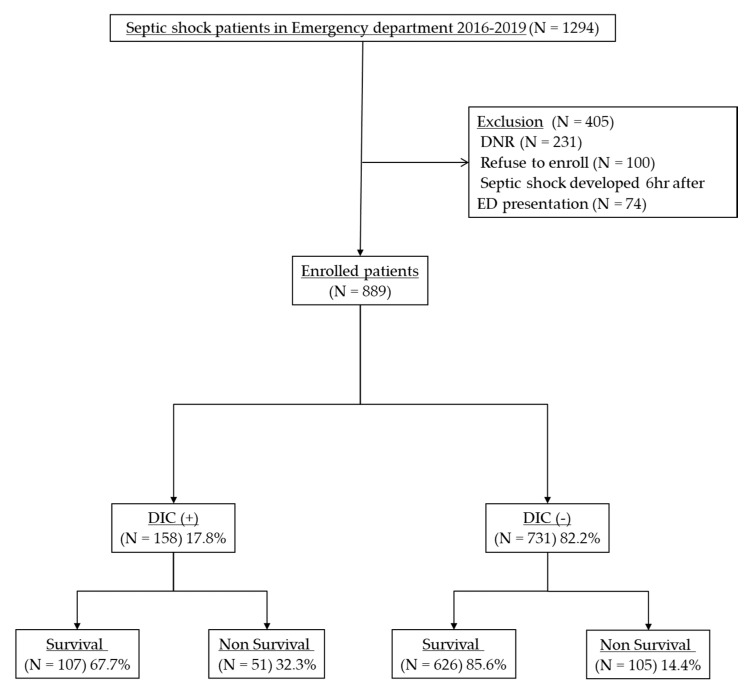
Patient flow diagram. TEG, thromboelastography; DNR, do not resuscitate; ED, emergency department; DIC, disseminated intravascular coagulation.

Using registry data, we analyzed 1294 patients with septic shock who had undergone TEG at admission. We excluded 231 patients with “do not attempt resuscitation” orders, 100 patients who refused to enroll in the study, and 74 patients who developed septic shock after 6 h of ED presentation. Of the 889 enrolled patients, 158 (17.8%) patients with septic shock were diagnosed with overt DIC with an ISTH score of ≥ 5 (Figure 1).

Table 1 presents the baseline characteristics, including demographic, clinical, and laboratory data of the DIC and non-DIC groups. The mean age of the non-DIC group was significantly higher than that of the DIC group, whereas we found no sex-related differences between the groups. Further, we did not observe significant differences in the previous medical history, except chronic liver disease, which was more frequent in the DIC group than in the non-DIC group. There were no significant differences in the source of infection between the groups, except blood stream infection, which was more frequent in the DIC group than in the non-DIC group (10.8% vs. 5.6%; *p* = 0.031). The DIC group had significantly lower levels of hemoglobin and albumin than the non-DIC group. The initial lactate levels were significantly higher in the DIC group than in the non-DIC group. Compared with the non-DIC group, the DIC group had significantly higher initial SOFA and APACHE II scores. The overall 28-day mortality was 17.5%; it was higher in the DIC group than in the non-DIC group (32.3% vs. 14.4%; *p* = 0.000).

Table 2 presents the coagulation profiles for each group. We observed significant differences between the DIC and non-DIC groups in terms of conventional coagulation test findings (including platelet count, PT (INR), and aPTT), fibrin-degradation product levels (D-dimer and FDP levels), and fibrinogen levels. The overall median ISTH score was 3 (interquartile range (IQR), 2–4); it was higher in the DIC group than in the non-DIC group (5 (IQR: 5–6) vs. 2 (IQR: 1–3); *p* = 0.000).

### 3.2. Thromboelastography Analysis

**Figure 2:**
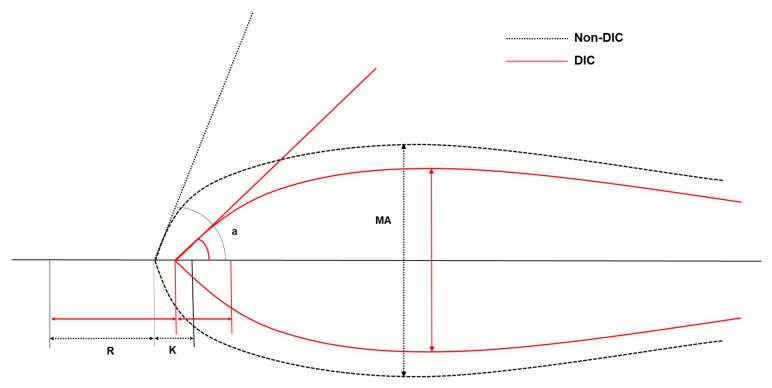
Graphical representation of thromboelastographic tracings and parameters of non-DIC (black dotted line), DIC (red solid line). R, reaction time; K, kinetic time; MA, maximal amplitude; a, alpha angle

Table 3 presents the TEG parameters for each group. All TEG values, except LY30, were significantly different between the DIC and non-DIC groups. The CI value was significantly higher in the non-DIC group than in the DIC group. Figure 2 shows a graphical representation of the TEG results for each group. The K, MA, and CI TEG values were discriminating, with AUCs > 0.750. The optimal TEG cutoff values for predicting DIC were K > 2.0 min, MA < 60 mm, and CI < 1.8.

The sensitivities, specificities, PPVs, NPVs, PLRs, and NLRs of the TEG values are shown in Table 4. Among the TEG values, MA < 60 showed the highest sensitivity at 79.11%, with a specificity of 73.05%, and an NPV of 94.18%.

### 3.3. Risk Factors Associated with DIC

The candidate risk factors associated with DIC included the following: age, initial lactate level, SOFA score, APACHE II score, K > 2.0 min, MA < 60 mm, and CI < 1.8 (Table 5). In multivariate logistic regression analysis, age, initial lactate level, SOFA score, and MA < 60 were independently associated with DIC.

## 4. Discussion

In this study, TEG profiles demonstrated significant differences among patients with septic shock in the DIC and non-DIC groups. Among the TEG values, MA < 60 was an independent factor associated with an increased risk for DIC (OR 5.616 (95% CI, 3.213–9.818)), with a sensitivity of 79%, specificity of 73%, and NPV of 94%.

DIC is defined as the systemic intravascular activation of coagulation arising from different causes [5]. Although the scoring system released by the ISTH in 2001 is a global standard for DIC diagnosis, there are concerns regarding diagnostic delays and unclear cutoff values for fibrin-related markers [6]. Therefore, several new diagnostic criteria were developed to overcome the drawbacks of the ISTH scoring system such as the Japanese Association for Acute Medicine (JAAM) DIC (2006) [23] and the Sepsis-Induced Coagulopathy (SIC) (2017) [24]. However, these new criteria have limited usefulness because they incorporate conventional coagulation assays, such as platelet counts and prothrombin time, which generally require at least 1 h to obtain results [25]. In contrast, TEG measures different aspects of the coagulation process in real time and may be performed rapidly at the patient’s bedside [26]. Sharma et al. reported that using TEG values to score patients may identify underlying disorders that could provoke overt DIC [27]. Koami et al. evaluated the association between TEG and DIC in 13 patients and reported that TEG profiles can be reliable indicators for sepsis-induced DIC [15]. However, the results of the abovementioned studies are not generalizable, since they included a very small number of patients. The present study incorporated a large sample of patients with septic shock, to overcome the limitations of prior studies, and demonstrated the predictive power of TEG profiles for overt DIC.

The manifestations of coagulopathy in sepsis vary from normal to hypercoagulability, hyperfibrinolysis, and hypocoagulability [28,29]. In the current study, the TEG profiles of the DIC group demonstrated a trend toward hypocoagulation (prolonged R and K and decreased MA and alpha angle). This result is consistent with that of a previous study investigating TEG in patients with severe sepsis, which reported that hypocoagulability was more often found in patients with overt DIC, whereas hypercoagulability was more often found in those without overt DIC [30]. In addition, Daudel et al. reported that coagulopathy progresses from hypercoagulable to hypocoagulable with increasing disease severity [29]. Therefore, as our study only included patients who had already developed shock (indicating that they were not in the early stage of the disease), hypocoagulability was more frequently observed. Moreover, another explanation is that there could be a possibility that patients who represent hypocoagulable presented to ED after the initial phase of hypercoagulable phase.

In our study, there was no difference in LY30 between the groups and mainly within the normal range (0~8%). Panigada et al. reported that impairment of fibrinolysis is associated with worse outcomes in sepsis [31]. As our study only included sepsis patients who developed shock, hyperfibrinolysis could be rarely observed. Moreover, our result coincided with the previous study, which evaluated severe sepsis patients who reported no difference in fibrinolysis activity between the overt DIC group and without the overt DIC group [30].

Sepsis-associated DIC is more frequently associated with organ dysfunction than with bleeding, which is a frequent complication of DIC with hematologic disorder [32]. In this study, indicators of organ dysfunction, such as initial lactate levels and SOFA and APACHE II scores, were significantly higher in the DIC group than in the non-DIC group. In the multivariate analysis, they were independent risk factors for DIC. Additionally, in our study, the 28-day mortality was significantly higher in the DIC group than in the non-DIC group (32.3% vs. 14.4%; *p* = 0.000), which is similar to the results of a previous study reporting that 32.5% patients with sepsis and overt DIC died [33].

Recently, efforts have been initiated to discover potential therapeutic targets to modulate pathophysiological pathways, such as coagulation systems, that may lead to organ dysfunction. Accordingly, studies have been conducted that incorporate several interventions to treat coagulopathy in patients with sepsis, including administration of anticoagulant supplements such as antithrombin [34,35], activated protein C (drotrecogin α) [36], and recombinant thrombomodulin [37]; however, the results were not optimistic. However, the subgroup analysis of patients with DIC showed that antithrombin treatment significantly improved prognosis [38]. According to recent studies, appropriate selection of patients and early and rapid diagnoses are essential for anticoagulant treatment [39,40]. Based on its ability to predict DIC, TEG could be helpful in the selection of appropriate candidates for coagulopathy treatment. Nevertheless, a future prospective multicenter study is required to elucidate the role of TEG as a predictor of DIC in septic-shock patients.

This study has several limitations. First, it did not necessarily reflect the general population because it was a single-center study, although it included a large sample of patients. Second, there were large numbers of patients excluded due to a do not resuscitate (DNR) order. As DNR is generally made in patients with critical illness with severe comorbidity, it could lessen the severity of the population. Third, studies have shown that TEG has a relatively broad reference range, and therefore, it might best be used to monitor changes within individuals [41,42]. If TEG values prior to septic shock onset were available for patients who eventually developed septic shock, the changes within these individuals could provide valuable information. Fourth, the timing of conventional coagulation and TEG assays was not coincident. Bias may have been introduced despite the exclusion of patients who developed septic shock after 6 h of ER presentation. Lastly, there is a possibility that patients in the non-DIC group could have progressed to DIC after admission. If consecutive data on daily ISTH scores and TEG profiles were available, the association between TEG and DIC might be more reliably demonstrated.

## 5. Conclusions

Early diagnosis of coagulopathy and initiation of treatment is crucial for patients with sepsis. Considering that TEG results are rapidly available at bedsides, MA < 60 could be a valuable tool for DIC prediction in patients with septic shock.

## Figures and Tables

**Table 1:** Baseline characteristics and outcomes of the study population.

Characteristics	Total (*N* = 889)	Non-DIC (*N* = 731)	DIC (*N* = 158)	*p*-Value
Age, years	65.6 ± 12.7	66.2 ± 12.5	62.8 ± 13.2	0.002
Male	521 (58.6)	437 (59.8)	84 (53.2)	0.131
Medical history				
Hypertension	314 (35.3)	56 (35.4)	258 (35.3)	1.000
Diabetes mellitus	227 (25.5)	196 (26.8)	31 (19.6)	0.070
Chronic pulmonary disease	89 (10.0)	76 (10.4)	13 (8.2)	0.467
Hematologic disease	55 (6.2)	44 (6.0)	11 (7.0)	0.715
Malignancy	425 (47.8)	343 (46.9)	82 (51.9)	0.292
Chronic renal disease	59 (6.6)	48 (6.6)	11 (7.0)	0.860
Chronic liver disease	141 (15.9)	86 (11.8)	55 (34.8)	0.000
Source of infection				
Pulmonary	216 (24.3)	187 (25.6)	29 (18.4)	0.065
Genitourinary	125 (14.1)	105 (14.4)	20 (12.7)	0.616
Gastrointestinal	124 (13.9)	99 (13.5)	25 (15.8)	0.449
Hepatobiliary	302 (34.0)	242 (33.1)	60 (38.0)	0.266
Blood stream	58 (6.5)	41 (5.6)	17 (10.8)	0.031
Others	36 (4.0)	28 (3.8)	8 (5.1)	0.503
Unknown	90 (10.1)	69 (9.4)	21 (13.3)	0.147
Laboratory findings				
White blood cell, ×10^3^/uL	11.8 ± 10.7	12.1 ± 10.8	10.7 ± 10.3	0.138
Hemoglobin, g/dL	10.7 ± 2.4	10.8 ± 2.4	10.1 ± 2.3	0.001
Blood urea nitrogen, mg/dL	31.1 ± 20.3	30.8 ± 20.8	32.7 ± 17.7	0.299
Creatinine, mg/dL	2.0 ± 2.3	1.9 ± 2.0	2.4 ± 3.1	0.053
Albumin, g/dL	2.7 ± 1.6	2.8 ± 1.6	2.4 ± 1.6	0.025
Initial Lactate, mmol/L	4.1 ± 3.1	3.7 ± 2.8	5.8 ± 3.8	0.000
Initial SOFA score	5.9 ± 3.1	5.4 ± 2.9	8.1 ± 3.0	0.000
APACHE II score	17.5 ± 8.1	16.8 ± 7.7	20.4 ± 9.1	0.000
Length of ICU stay	8.1 ± 11.1	8.1 ± 11.7	8.0 ± 9.0	0.924
Clinical Outcome				
28-day mortality	156 (17.5%)	105 (14.4%)	51 (32.3%)	0.000
90-day mortality	273 (30.7%)	195 (26.7%)	78 (49.4%)	0.000

Values are expressed as the mean ± standard deviation, median (interquartile range), or number (%). SOFA, Sequential Organ Failure Assessment; APACHE, Acute Physiology and Chronic Health Evaluation; ICU, intensive care unit.

**Table 2:** Comparison of the coagulation profile in each group.

	Total (*N* = 889)	Non-DIC (*N* = 731)	DIC (*N* = 158)	*p*-Value
Platelet, ×10^3^/uL	161.9 ± 114.1	180.9 ± 112.3	74.2 ± 75.0	0.000
PT (INR)	1.47 ± 0.91	1.34 ± 0.53	2.05 ± 1.74	0.000
aPTT, s	34.5 ± 15.9	32.1 ± 11.7	45.7 ± 25.3	0.000
D-dimer, ug/mL	4.6 (2.2–10.6)	3.8 (1.8–7.3)	12.3 (7.7–21.8)	0.000
FDP, ug/mL	13.9 (7.2–26.6)	12.1 (6.8–21.2)	29.6 (17.7–54.4)	0.000
Fibrinogen, mg/dL	424.5 ± 186.5	446.3 ± 180.5	323.3 ± 180.7	0.000
ISTH score	3 (2–4)	2 (1–3)	5 (5–6)	0.000

Values are expressed as the mean ± standard deviation, the median (interquartile range), or number (%). PT, prothrombin time; INR, international normalized ratio; aPTT, activated partial thromboplastin time; FDP, fibrinogen degradation production; ISTH, International Society on Thrombosis and Hemostasis.

**Table 3:** Comparison of the thromboelastography parameters in each group.

Variables	Total (*N* = 889)	Non-DIC (*N* = 731)	DIC (*N* = 158)	AUC	*p*-Value
R, min	6.9 ± 6.7	6.5 ± 5.9	9.0 ± 9.5	0.679	0.001
K, min	2.6 ± 3.9	2.2 ± 3.4	4.3 ± 5.3	0.793	0.000
Alpha angle, degree	61.6 ± 15.8	64.0 ± 14.5	50.6 ± 16.9	0.766	0.000
MA, mm	61.3 ± 14.5	64.1 ± 12.9	48.5 ± 14.6	0.814	0.000
LY30, %	1.2 ± 6.2	1.3 ± 6.0	0.9 ± 7.1	0.631	0.549
Coagulation index	2.2 ± 2.5	2.7 ± 2.2	−0.1 ± 2.9	0.819	0.000

Values are expressed as the mean ± standard deviation. AUC, area under the curve; R, reaction time; K, kinetic time; MA, maximal amplitude; LY30, lysis after 30 min.

**Table 4:** Performance parameters to predict disseminated intravascular coagulation, using the cutoff value of thromboelastography in the study population.

Variables	Non-DIC	DIC	Se/Sp (%)	PPV/NPV (%)	PLR/NLR
K > 2.0, min	235 (32.1%)	122 (77.2%)	77.22/67.85%	34.17/93.23%	2.40/0.34
MA < 60, mm	77 (10.5%)	80 (50.6%)	79.11/73.05%	38.82/94.18%	2.94/0.29
CI < 1.8	187 (25.6%)	120 (75.9%)	75.95/74.42%	39.09/93.47%	2.97/0.32

Values are expressed as number (%). K, kinetic time; MA, maximal amplitude; CI, coagulation index; DIC, disseminated intravascular coagulation; Se, sensitivity; Sp, specificity; PPV, positive predictive value; NPV, negative predictive value; PLR, positive likelihood ratio; NLR, negative likelihood ratio.

**Table 5:** Univariate and multivariate analysis for predicting disseminated intravascular coagulation.

Characteristics	Univariate OR (95% CI)	Multivariate OR (95% CI)	*p*-Value
Age, years	OR, 0.979 (95% CI, 0.967–0.992)	OR, 0.981 (95% CI, 0.965–0.997)	0.019
Initial lactate, mmol/L	OR, 1.210 (95% CI, 1.150–1.273)	OR, 1.179 (95% CI, 1.109–1.253)	0.000
SOFA score	OR, 1.316 (95% CI, 1.241–1.397)	OR, 1.167 (95% CI, 1.091–1.248)	0.000
APACHE II score	OR, 1.052 (95% CI, 1.031–1.074)		
K > 2.0, min	OR, 7.153 (95% CI, 4.781–10.701)	OR, 1.696 (95% CI, 0.972–2.959)	0.063
MA < 60, mm	OR, 10.268 (95% CI, 6.767–15.579)	OR, 5.616 (95% CI, 3.213–9.818)	0.000
CI < 1.8	OR, 9.187 (95% CI, 6.153–13.717)		

Multivariate analysis included logistic regression analysis and backward elimination. OR, odds ratio; CI, confidential interval; SOFA, Sequential Organ Failure Assessment; APACHE, Acute Physiology and Chronic Health Evaluation; K, kinetic time; MA, maximal amplitude; CI, coagulation index.

## Supplementary Materials

The following are available online at https://www.mdpi.com/2077-0383/9/12/3883/s1. Table S1: Scoring system for overt DIC proposed by ISTH.
