# Supplementary material for: Role of Thromboelastography as an Early Predictor of Disseminated Intravascular Coagulation in Patients with Septic Shock
Source: J Clin Med. 2020 Nov 29;9(12):3883. doi: 10.3390/jcm9123883 (PMC7760761; doi:10.3390/jcm9123883)
Supplement: Supplementary file 1 [file jcm-09-03883-s001.pdf]

Supplementary Table S1. Scoring system for overt DIC proposed by ISTH

Score	
**Platelet count (x 10^3/uL)**	
< 50	2
≥ 50 and < 100	1
≥ 100	0
**Fibrin-related markers**	
Strong increase	3
Moderate increased	2
No increase	0
**Prothrombin time (s)**	
≥ 6	2
3-6	1
< 3	0
**Fibrinogen level (g/mL)**	
< 100	1
≥ 100	0
**Calculate score**	
If ≥ 5, compatible with over DIC	
If < 5, suggestive for non-overt DIC	

DIC; disseminated intravascular coagulation; ISTH, international society of thrombosis and hemostasis
